# *RUNX1* mutation has no prognostic significance in paediatric AML: a retrospective study of the AML-BFM study group

**DOI:** 10.1038/s41375-023-01919-8

**Published:** 2023-05-15

**Authors:** Stephanie Sendker, Amani Awada, Sophia Domagalla, Michael Sendker, Eser Orhan, Lina Marie Hoffmeister, Evangelia Antoniou, Naghmeh Niktoreh, Dirk Reinhardt, Nils von Neuhoff, Markus Schneider

**Affiliations:** 1 Department of Paediatric Hematology and Oncology, University Children’s Hospital Essen, University of Duisburg-Essen, 45147 Essen, Germany; 2 University of Applied Sciences for Economics and Management (FOM), 20357 Hamburg, Germany; 3 Centre for Research Acceleration in Paediatrics GmbH, 30175 Hannover, Germany

**Keywords:** Acute myeloid leukaemia, Acute myeloid leukaemia

## Abstract

In acute myeloid leukaemia (AML) *RUNX1* mutation is characterised by certain clinicopathological features with poor prognosis and adverse risk by the European LeukemiaNet recommendation. Though initially considered as provisional category, the recent World Health Organisation (WHO) classification of 2022 removed *RUNX1*-mutated AML from the unique entity. However, the significance of *RUNX1* mutation in paediatric AML remains unclear. We retrospectively analysed a German cohort of 488 paediatric patients with de novo AML, enroled in the AMLR12 or AMLR17 registry of the AML-BFM Study Group (Essen, Germany). A total of 23 paediatric AML patients (4.7%) harboured *RUNX1* mutations, 18 of which (78%) had *RUNX1* mutation at initial diagnosis. *RUNX1* mutations were associated with older age, male gender, number of coexisting alterations and presence of *FLT3*-ITD but mutually exclusive of *KRAS*, *KIT* and *NPM1* mutation. *RUNX1* mutations did not prognostically impact overall or event-free survival. Response rates did not differ between patients with and without *RUNX1* mutations. This comprehensive study, comprising the largest analysis of *RUNX1* mutation in a paediatric cohort to date, reveals distinct but not unique clinicopathologic features, with no prognostic significance of *RUNX1*-mutated paediatric AML. These results broaden the perspective on the relevance of *RUNX1* alterations in leukaemogenesis in AML.

## Introduction

Acute myeloid leukaemia (AML) is a genetically heterogeneous disease. Analyses of genetic aberrations helped to unravel a complex genetic landscape and improved our knowledge of AML pathogenesis, leading to refined, risk-adapted treatment allocation [1–4]. In paediatric AML, optimising genetic and response-dependent risk-group stratification, allocating high-risk patients to receive allogeneic stem cell transplantation (SCT) in first complete remission (CR) allows survival rates of about 80% [4]. Notably, frequency of several prognostic biomarkers differs greatly between adult and paediatric AML, suggesting a different ontogeny in childhood AML [5–7]. Moreover, distinct age-specific genetic profiles point at a unique biology in paediatric AML and implicate the necessity for precise, age-tailored diagnostic and therapeutic approaches [7–11].

Intragenic mutations of the runt-related transcription factor-1 (*RUNX1*) have been associated with distinct clinical and genetic features as well as inferior prognosis in adult AML [12–16]. These results prompted the inclusion of “AML with mutated *RUNX1*” as new provisional entity of the World Health Organisation (WHO) classification as part of the revision in 2016 [17, 18]. Due to lack of specificity, this was rejected by the latest 5^th^ edition of the WHO classification [19]. Considering the independent association with unfavourable risk, the European LeukemiaNet (ELN) recommendation classified *RUNX1*-mutated AML into the adverse risk category [20, 21].

The RUNX1 transcription factor, encoded on chromosome 21q22.3, is crucial for early lineage differentiation and indispensable for definite hematopoiesis [22, 23]. In AML, the core-binding factor (CBF) family member *RUNX1* is involved in the recurrent chromosomal aberrations t(8;21), leading to the leukaemia-initiating *RUNX1*::*RUNX1T1* fusion, predicting favourable prognosis [24]. Native *RUNX1* was found to critically promote leukaemogenesis in t(8;21) AML [25].

Intragenic mutations of the *RUNX1* gene were preliminary reported in MDS, secondary and therapy-related AML, radiation exposed myelodysplastic syndrome (MDS) and AML [26–28]. Inherited *RUNX1* mutation were found to cause familial platelet disorder (FPD) with a propensity to MDS and AML [29–31]. To date, the impact of the RUNX1 mutation in paediatric AML has remained unclear. There are only a few controversial reports, with a limited data and number of patients on the *RUNX1* mutation in paediatric AML [32–35].

This retrospective study elucidates the pathological and clinical implication of *RUNX1* mutation in a substantial paediatric AML cohort. By targeted sequencing, we assess concomitant genetic changes and their impact in development of paediatric AML.

## Methods

### Study participants

A paediatric cohort of 488 patients (0–18 years), diagnosed with de-novo AML between 2015 and 2021 was included. The cohort represents a German paediatric AML population, excluding patients with acute promyelocytic leukaemia (FAB M3), Down syndrome (ML-DS), secondary and treatment-related AML. All included patients were enroled and treated either according to the AMLR12 registry (EudraCT number: 2013-000018-39) or AMLR17 registry (DRKS number: DRKS00013030) of the AML-BFM Study Group. The ethical committees and institutional review boards of the university hospital of Hannover and Essen approved both registries according to the declaration of Helsinki. Informed consent was obtained from patients and their legal guardians prior to treatment participation. Initial and follow-up diagnostics including morphological, immunophenotypic and molecular genetic testing were performed centrally by the German AML-BFM reference laboratory Essen according to standard procedures. Analysis for classical cytogenetics and fluorescence in situ hybridisation (FISH) were carried out by the Institute of Human Genetics in Hannover Medical School. The treatment recommendations of analysed registries included similar cytarabine- and daunorubicin-based induction chemotherapy [36]. Depending on the risk stratification intensive chemotherapy, maintenance therapy and allogeneic SCT were performed by indication.

### Genetic analyses

Primary bone marrow or peripheral blood samples from paediatric patients were subjected to molecular genetic screening at the time of diagnosis by next generation sequencing (NGS) using the TruSight® Myeloid Panel (Illumina, San Diego, California, USA). Using the standard NGS technology, the mutational screening included 54 different leukaemia-associated genes (Supplementary Table S2b). Sequencing was performed on the Illumina MiSeq™DX System (Illumina, San Diego, California, USA). Analysis was conducted using the SOPHIA DDM^TM^ software (Sophia Genetics, Switzerland). *RUNX1* mutations were confirmed by PCR and subsequent Sanger sequencing.

To check for germline mutations, paired remission samples of *RUNX1*-mutated AML patients, harbouring a VAF above 30 were analysed by PCR to detect the respective *RUNX1* mutation.

RNA sequencing was analysed using the TruSight® RNA Fusion Panel (Illumina, San Diego, CA, USA) according to manufacturer’s recommendations. Classical karyotyping was performed following the International System for Human Cytogenetic (International System of Cytogenetic Nomenclature; ISCN 1995–2013).

### Statistical analysis

Patient characteristics between groups were compared using Pearson chi-square or Fisher’s exact test for categorical variables and Mann–Whitney U test for continuous variables. A *p*-value of less than 0.05 was considered to indicate statistical significance. Survival analysis included Overall survival (OS), defined as the time from diagnosis to death or last follow-up, Event-free survival (EFS), defined as the time from diagnosis to relapse, secondary malignancy, death, or the time of last follow-up. Kaplan–Meier curves were calculated for OS and EFS. The log-rank test was obtained to compare survival differences. To identify prognostic variables, Cox-models were used for univariate and multivariate regression analyses. Results were reported as hazard risk ratios with 95% confidence intervals (CI) indicated. Following complexity and dimensionality reduction to a reasonable, recommended number of 50 principal components (PCs), the unsupervised clustered data were visualised using t-distributed stochastic neighbourhood embedding (t-SNE) according to recent recommendations [37] using Seaborn and Plotly Express Python visualisation libraries based on matplotlib (Python 3.9.13, Jupyter Notebook 6.4.12). Statistical analysis was conducted using SPSS software version 28.0 (Chicago, IL, USA) and R software version 4.0.2 (http://www.Rproject.org). Illustrations were generated using Graphpad Prism™ 8.3, R software version 4.0.2, Python 3.9.13 and DOG 2.0 [38].

## Results

### Characteristics of *RUNX1* mutations

**Fig. 1:**
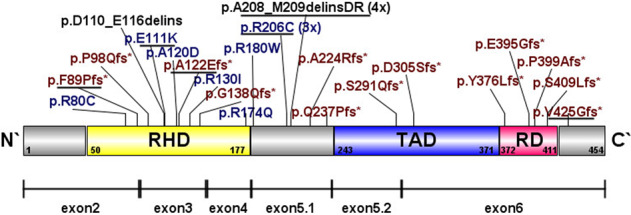
Distribution of *RUNX1* mutations in paediatric AML. Gene diagram depicting the structure of *RUNX1* (Ref Seq NM_001001890). *RUNX1* mutations are visualised and assigned to the corresponding locus. Six mutations were detected in five relapsed samples (black line). RHD Runt homology domain, TAD transactivation domain, RD repression domain. Protein in the top row and mRNA of isoform 1b, variant 2 in the bottom row (NM_001001890.3, reference sequence).

In 23 patients (4.7%), 27 *RUNX1* mutations (OMIM No. 151385) were detected of which 24 mutations were distinct from each other (Fig. 1, Supplementary Table S1a, b). Within the *RUNX1*-mutated cohort, 18 out of 23 patients presented at least one *RUNX1* mutation at initial diagnosis (78%). Among eight patient-matched paired diagnosis and relapse samples, *RUNX1* mutations were detectable only at relapse in five cases (Supplementary Tables S1a and S6). The same *RUNX1* mutation was present in two patients at both time points. In one case, *RUNX1* mutation was undetectable at relapse. Comparing the paired samples in this case, two additional mutations were lost from initial diagnosis to relapse. Regarding morphologic, immunophenotypic and cytogenetic criteria the early relapse resembled the initial diagnosis, indicating that the loss of the *RUNX1* mutation occurred during the process of clonal evolution (Supplementary Tables S6).

Considering all 27 detected *RUNX1* coding sequence alterations, the majority were characterised as frameshift (48.1%), followed by nine missense (33.3%) and two different deletion-insertions (delins) in five cases (18.5%). Four patients were found to have two different *RUNX1* mutations, three of which shared the same combinatorial pattern (p.A208_M209delinsDR, p.R206C), including the sample with *RUNX1* mutation only at diagnosis. Among the five relapsed samples harbouring *RUNX1* mutation only at relapse, three frameshifts, two missenses, and the repeatedly occurring mutational combination were found. Considering the distribution on the *RUNX1* gene (Fig. 1), one mutation was allocated to exon 2, six mutations to exon 3, one to exon 4, seven to exon 5.1, (including p.A208_M209delinsDR, p.R206C), one to exon 5.2 and five to exon 6. Mutations were assigned to different functional groups, whilst the largest proportion of eleven mutations (40.7%) was clustered in the runt homology domain (RHD), two mutations (7.4%) were in the transactivation domain (TAD), four (14.8%) in the repression domain (RD) and eight (29.6%) mutations were located between the TAD and RD. One frameshift mutation was localised at C-terminal to the RD. In the RHD all three different kinds of mutations were discovered, whereas in the TAD and RD only frameshift mutation occurred. To exclude germline mutations and proof the somatic origins, *RUNX1-*mutated AML with a variant allele frequency (VAF) > 30% were selected for germline-testing. Since one of which never reached remission, samples at remission were available for nine patients. The suspected *RUNX1* mutation was not detectable in any of the remission samples, confirming the somatic origin.

### Association with clinical characteristics

**Fig. 2:**
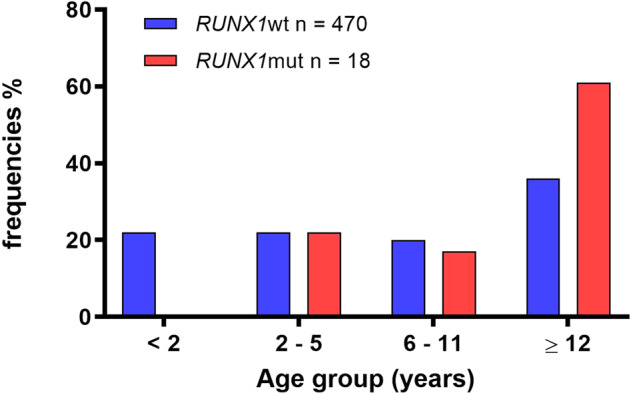
Distribution of *RUNX1* mutation according to age groups in paediatric AML. Median age was higher in *RUNX1*-mutated (red bars) patients than in *RUNX1* wt (blue bars) patients. *RUNX1*mut *RUNX1*-mutated, *RUNX1*wt *RUNX1* wildtype.

Compared to *RUNX1* wildtype (wt) (*n* = 470), patients with *RUNX1*-mutated de novo AML (*n* = 18) were significantly older at diagnosis (11.7 [2–17] *vs*. 8.3 [0–18] years, *p* = 0.02), (Table 1). Age curve of mutated patients is clearly shifted to adolescent age (>12 years), whilst there is a relatively uniform age-distribution in *RUNX1* wt-patients, (Fig. 2). *RUNX1* mutations tends to associate with male gender (male: 50 vs. 72%, *p* = 0.06). In terms of the AML morphology, *RUNX1*-mutated were more likely to be myelomonocytic compared to non-mutated samples (FAB M4, 33 vs. 17%, *p* = 0.1), followed by AML with minimal differentiation and without maturation (FAB M0, 6 vs. 1%, *p* = 0.26; FAB M1, 22 vs. 17%, *p* = 0.29). In contrast, the number of samples evaluated as acute monocytic leukaemia (FAB M5, 11 vs. 23%, *p* = 0.39) and AML with maturation (FAB M2, 17 vs. 22%, *p* = 0.78) were lower in the mutated cohort. None of the six patients with acute erythroid leukaemia harboured *RUNX1* mutation. The *RUNX1*-mutated patient samples showed no significant difference in terms of median haemoglobin level, white blood cell, platelet, bone marrow and peripheral blast count at diagnosis. Involvement of the central nervous system (CNS) at diagnosis was almost equal in both groups.

**Table 1:** Comparison of demographic and clinical characteristics of *RUNX1*-mutatedand wildtype paediatric patients with AML.

Characteristics	*RUNX1*wt (*n* = 470)	*RUNX1*mut (*n* = 18)	*p*-value
Gender, male, *n* (%)	235 (50)	13 (72)	0.07
Age, years			
Median (range)	8 (0–18)	11 (2–17)	0.02
FAB classification, *n* (%)			
M0	7 (1)	1 (6)	0.26
M1	63 (13)	4 (22)	0.29
M2	105 (22)	3 (17)	0.78
M4	81 (17)	6 (33)	0.11
M4 eo	39 (8)	1 (6)	1.0
M5	109 (23)	2 (11)	0.39
M6	6 (1)	0 (0)	1.0
M7	34 (7)	1 (6)	1.0
n.a.	26	0	
CNS involvement, *n* (%)	42 (9)	2 (11)	0.67
WBC count, 10^9/l			
Median (range)	29.8 (0.135–394)	34.2 (0.87–163)	0.24
n.a.	102	0	
Platelet count, 10^9/l			
Median (range)	91 (4–722)	105 (28–249)	0.82
n.a.	119	0	
Haemoglobin, g/dl			
Median (range)	8.3 (2–18)	8.8 (6–14)	0.34
n.a.	50	0	
Bone marrow blasts, %			
Median (range)	64 (5–100)	65 (22–97)	0.92
n.a.	71	1	
Peripheral blood blasts, %			
Median (range)	45 (0–99)	41 (0–98)	0.23
n.a.	166	0	
Risk stratification, *n* (%)			
Standard risk	119 (25)	4 (22)	0.79
Intermediate risk	197 (42)	10 (56)	0.47
High risk	119 (25)	4 (22)	0.79
n.a.	35 (7)	0	

*Mut* mutation, *Wt* wildtype, *FAB* French-American-British, *CNS* central nervous system, *wbc* white blood count.

### Association with genetic characteristics

Cytogenetic data were available for all 18 *RUNX1*-mutated and 460 (98%) *RUNX1 wt*-patients (Table 2). Prevalence of normal and complex karyotype did not differ significantly between *RUNX1*-mutated and wt-cohort (44 vs. 26%, *p* = 0.11 and 22 vs. 17%, *p* = 0.55, respectively). Frequent cytogenetic aberrations accompanying the *RUNX1* mutation were t(8;21) and trisomy +8, showing almost equal distribution (17 vs. 12%, *p* = 0.7 and 11 vs. 15%, *p* = 1.0). *RUNX1* mutation tends to associate with −7/7q, (17 vs. 5%, *p* = 0.08). *RUNX1* mutation did not co-occur together with *KMT2A* rearrangement (KMT2Ar), (0 vs. 19%, *p* = 0.04). The recurrent aberration inv(16)/t(16;16) was present in one patient sample (6 vs. 7%, *p* = 0.82). In addition, *RUNX1* mutations did not occur together with other recurrent cytogenetic changes, namely the t(6;9), −5/5q, trisomy +11 or +13.

**Table 2:** Correlation of *RUNX1* mutation with cytogenetic aberrations in paediatric AML.

	*RUNX1*wt (*n* = 470)	*RUNX1*mut (*n* = 18)	*p*-value
Cytogenetic data			
Normal KT, *n* (%)	124 (26)	8 (44)	0.11
Complex KT, *n* (%)	82 (17)	4 (22)	0.55
Nummeric aberration, *n* (%)	131 (28)	5 (28)	0.91
t(8;21), *n* (%)	58 (12)	3 (17)	0.72
inv16/t(16;16), *n* (%)	35 (7)	1 (6)	1.0
t(9;11), *n* (%)	37 (8)	0	0.38
t(6;9), *n* (%)	5 (1)	0	1.0
t(1;22), *n* (%)	4 (1)	0	1.0
inv3/t(3;3), *n* (%)	5 (1)	1 (6)	0.18
+8, *n* (%)	71 (15)	2 (11)	1.0
+11, *n* (%)	4 (1)	0	1.0
+13, *n* (%)	7 (1)	0	1.0
−5/5q, *n* (%)	11 (2)	0	1.0
−7/7q, *n* (%)	25 (5)	3 (17)	0.08
KMT2Ar, *n* (%)	90 (19)	0	0.04
NUP98/NSD1, *n* (%)	5 (1)	1 (6)	0.18
Missing data, *n* (%)	11 (2)	0	

*KT* karyotype, *KMT2Ar* KMT2A rearrangement

**Fig. 3:**
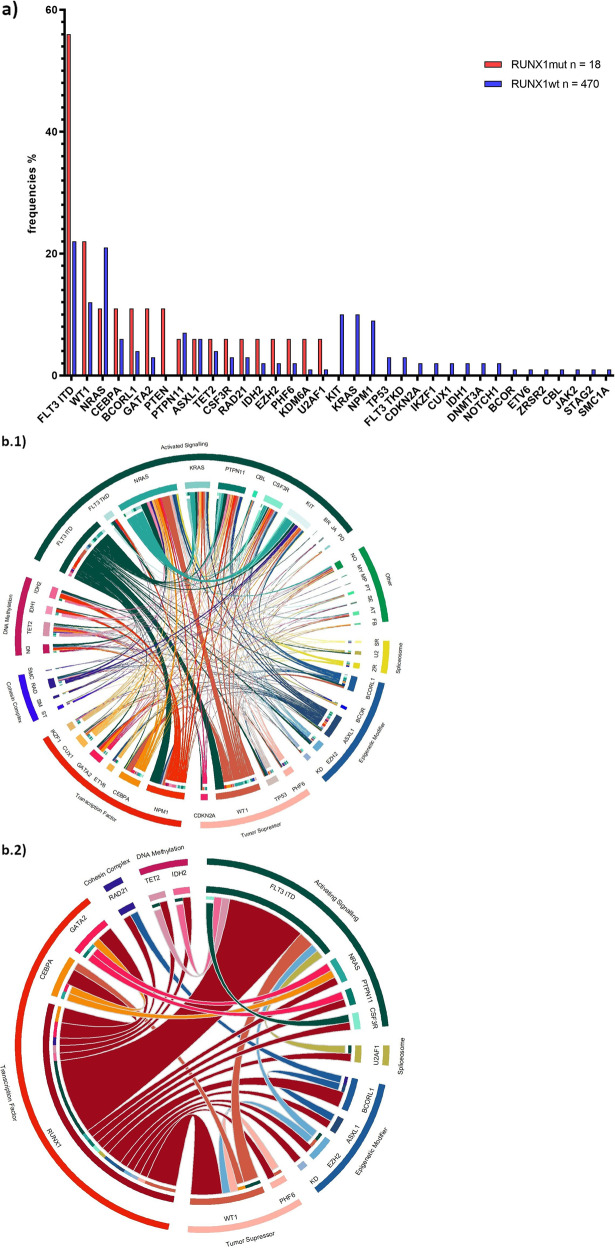
Distribution of genetic mutation according to *RUNX1* mutation in paediatric AML patients. **a** Frequency of molecular genetic mutations as listed on the x-axis in *RUNX1*-mutated patients (red bars) and wt-patients (blue bars). **b** Chord diagram displaying the distribution and mutational pattern of molecular genetic aberrations in *RUNX1-*wt cohort (b.1) and *RUNX1*-mutated cohort (b.2) in childhood AML. SR *SRSF2*, U2 *U2AF1*, SF *SF3B1*, ZR *ZRSR2*, KD *KDM6A*, NU *NUP98::NSD1*, KM *KMT2A*, ST *STAG2*, SM *SMC1A*, RAD *RAD21*, SMC *SMC3*, DN *DNMT3A*, BR *BRAF*, JA *JAK2;* PD *PDGFRA*, NO *NOTCH1*, MY *MYD88*, MP *MPL*, PT *PTEN*, SE *SETBP1*, AT *ATRX*, FB *FBXW7*.

Addressing the mutational spectrum of co-occurring mutations, all 18 *RUNX1*-mutated and 470 wt-cases were evaluated for 53 additional AML-related genes assayed by targeted NGS (Fig. 3, Supplementary Table S2b). The average number of concomitant aberrations was significantly increased in *RUNX1*-mutated patient cohort (2.4, range 0–5) compared with the wt-cohort (1.7, range 0–6), (*p* = 0.02). Whereas the largest proportion of wt-patients (32%) had one detectable mutation, most *RUNX1*-mutated cases (50 %) harboured two additional, accompanying mutations.

*RUNX1* mutation was correlated with the presence of *FLT3* with internal tandem duplication (*FLT3*-ITD), being the most common co-occurring genetic aberration in the *RUNX1*-mutated cohort (56 vs. 22%, *p* = 0.002). The second most frequent *RUNX1* accompanying mutation was in *Wilms-tumor1* (*WT1)* gene, which was found in 4 of the 18 *RUNX1*-mutated cases (22 vs. 12%, *p* = 0.26). Whereas *NRAS* mutations occurred as second most common mutations in wt-patients, it was rarely found in *RUNX1*-mutated samples (6 vs. 21%, *p* = 0.14). *CEBPA* aberrations were detected in two *RUNX1*-mutated samples and were slightly more commonly mutated in the *RUNX1-*mutated cohort (11 vs. 6%, *p* = 0.28). Upon further differentiation of all 27 *CEBPA*-mutant samples, *CEBPA* aberration was biallelic in 15 patients, including two *RUNX1*-mutated (11 vs. 3%, *p* = 0.11). *RUNX1* mutations were mutually exclusive of recurrent alterations in *KRAS, KIT* and *NPM1* (*p* = .024, *p* = 0.4 and *p* = 0.39). Other mutations studied were more evenly distributed in both cohorts, with no tendency for a specific correlation.

Genetic mutations detected by NGS were assigned to different functional groups according to the functional categorisation of the mutated genes [2] (Supplementary Table S2c). The largest proportion was attributed to activated signalling, which made up 41% among *RUNX1*-mutated and 50% among wt-cases. Mutations affiliated to epigenetic regulator occurred more frequently together with *RUNX1* mutation (22 vs 10%). Alterations of transcription factors, excluding *RUNX1*, were the second most common aberrations among wt-cases, showing comparable frequency in *RUNX1*-mutated and wt-cohort (13 vs. 15%). Aberrations of tumour suppressor genes were evenly distribution between *RUNX1*-mutated and wt-cohorts (16 *vs*. 12%). Together, there was no clear association between *RUNX1* mutations and any of the other genetic groups. Accordingly, *RUNX1*-mutated samples projected scattered without showing definable, separate clustering in the t-SNE plot, which contrasts with the well-known distinct entity of CBF in AML (Supplementary Fig. S1).

### Impact of *RUNX1* mutations on response, outcome and survival

Response to therapy was comparable between *RUNX1*-mutated and wt-cases (94 *vs*. 84% achieved CR, *p* = 0.33). None of *RUNX1*-mutated, but 20 of wt-cases suffered early death (4%) (Supplementary Table S3). The proportion of patients undergoing SCT in first CR was evenly distributed between the *RUNX1*-mutated, and wt-cohorts (23 vs. 22%). Likewise, number of patients transplanted as salvage therapy were almost equal in both groups (Supplementary Table S3).

**Fig. 4:**
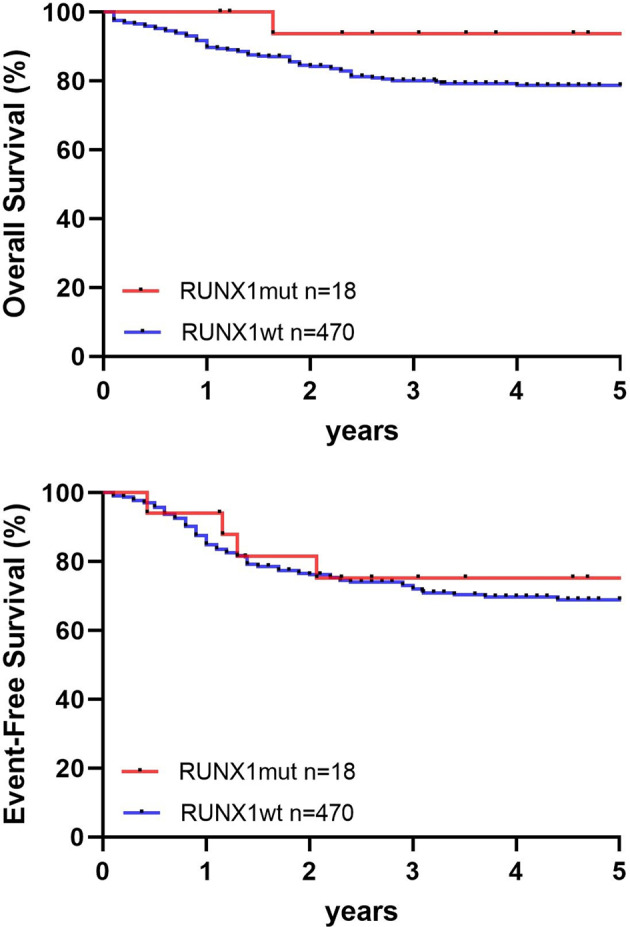
Survival based on *RUNX1* mutation status in paediatric AML. Data are shown for Overall Survival (OS) and Event-Free Survival (EFS). *RUNX1mut* *RUNX1*-mutated, *RUNX1wt* *RUNX1* wildtyp, *p*-value.

OS and EFS were compared between patients with and without *RUNX1* mutation (Fig. 4). In the entire cohort, the mean follow-up time of survival for patients alive was 5.9 ± 1.3 years (95% CI, 5.7–6.1). *RUNX1* mutations had no significant effect on 5-year OS (93 ± 6.1 *vs*. 78 ± 2.2%, *p* = .12) and EFS (55 ± 13.9 *vs*. 59 ± 2.6%, *p* = .53). Frequency of the associated outcome parameter death and relapse were comparable between *RUNX1*-mutated and wt-cohorts (Supplementary Table S3).

Univariable regression analysis revealed no strong prognostic impact of *RUNX1* mutation on OS (HR 0.43, *p* = 0.16) or EFS (HR 0.78, *p* = 0.55; Supplementary Table S4a). Multivariable models were created following adjustment for age, risk groups, *FLT3*-ITD, SCT and additional mutations (Supplementary Table S4b). *RUNX1* mutation was not predictive to experience any event (HR 1.4, *p* = 0.39) nor of death (HR 4.2 *p* = 0.17). Since *RUNX1* mutation was significantly associated with *FLT3*-ITD, increased patient age and number of mutations, subgroup analyses were performed to separately assess the relative prognostic relevance of *RUNX1* mutation within these subgroups (Supplementary Table S5a). In the presence of *FLT3*-ITD, concomitant *RUNX1* mutation did not impact OS (80 ± 17.9 vs. 78.8 ± 4.3, *p* = 0.42) or EFS (57.1 ± 16.4 vs. 56.6 ± 5.5, *p* = 0.68). In children younger than 12-years at diagnosis, *RUNX1*-mutated and wt patients showed comparable outcome (OS, 63.5 ± 16.9 vs. 77.7 ± 2.8, *p* = 0.58, EFS, 53.3 ± 17.3 vs. 62.3 ± 3.2, *p* = 0.95). In older paediatric patients (>12-years) *RUNX1* mutation tends to associate with increased OS (100 ± 0 vs. 76.9 ± 3.5, *p* = 0.11), but not EFS (71.6 ± 14 vs. 54.5 ± 4.3, *p* = 0.36). Among patients harbouring more than two additional genetic alterations, *RUNX1* mutation neither impact OS (90.9 ± 8.7 vs. 74.9 ± 5.0, *p* = 0.24), nor EFS (54.2 ± 16.2 vs. 49.3 ± 6.3, *p* = 0.48). When separately assessing impact of *RUNX1* mutation in the context of SCT, patients with and without *RUNX1* mutation, receiving SCT in first CR achieved comparable survival rates (OS, 75.0 ± 21.7 vs. 77.0 ± 4.4, *p* = 0.91, EFS 60 ± 21.9 vs. 57.1 ± 5.2, *p* = 0.98). Separate survival analyses, conducted for standard-, intermediate-, and high-risk groups revealed no strong difference of survival by *RUNX1* mutation for risk-associated groups (Supplementary Table S5b). Together, in our paediatric cohort, *RUNX1* mutation showed no prognostic significance.

## Discussion

AML with *RUNX1* mutation was considered as adverse-risk AML by the ELN and new provisional entity in the 2016 WHO classification given its characteristic clinicopathologic features and inferior outcome in adult AML [13, 16–18, 20]. In 2022, the preliminary stand-alone entity was removed due to lack of specificity [19]. In paediatric AML, large-scale sequencing studies suggested a distinct genetic landscape with clinical significance [7, 8, 11]. However, impact of *RUNX1*-mutated AML in paediatrics has largely remained obscure.

This retrospective study evaluates *RUNX1* mutations in the largest paediatric cohort to date. Prevalence of the studied *RUNX1*-mutated paediatric AML cases is relatively low (3.7%). In comparison, in adult AML, a frequency of ~5–13% has been reported, with highest prevalence when including elderly patients (>60 years) [13, 16, 39]. Accordingly, reported prevalence of *RUNX1* mutation was lower in younger patients and an association with patients age has been shown before [12–14, 33]. Likewise, in the paediatric cohort studied here, *RUNX1* mutation was found more frequently in older children. Consistent with previous findings our results confirm a positive correlation to male gender in paediatrics. Unlike previous studies [13, 15, 33], we could not detect a correlation with minimally differentiated AML (FAB M0). In AML with M0 phenotype, biallelic *RUNX1* mutations have been frequently described, where in case of heterozygous *RUNX1* mutation, a deletion on the other allele is suggested to result in a functional inactivation of *RUNX1* [31, 40]. However, this could not be confirmed in the current paediatric cohort, since there was only one heterozygous, biallelic *RUNX1* mutation in a FAB M4 sample, without evidence of further biallelic mutation.

The mutational spectrum revealed a higher proportion of frameshift mutations and a clustering of missense mutations at the N’-terminus, especially in RHD. A similar distribution of *RUNX1* mutation according to different functional domains and clustered mutations at the N´-terminus was previously reported in adult AML, whilst Greif *et al*. detect a greater proportion of missense mutation in cytogenetically normal AML [14–16]. Unexpectedly, in three patients we found a recurrent combination of the same missense and delins mutational pattern, which were not described before. These mutations had a low VAF (<10) and were only detectable at diagnosis or relapse in paired patient-matched samples, suggesting high mutational instability. Prevalence and implication of germline mutations in AML samples with *RUNX1* mutation has been discussed controversially [41, 42]. In our study, there was no evidence of germline mutations, which may not seem surprising given the reported wide age range for AML onset in FPD [30, 31]. Interestingly, in the current cohort, a child with Down Syndrome related AML (ML-DS) who was excluded a-priori from the analysis, harboured the same *RUNX1* mutation in remission, suggesting a germline mutation (data not shown). To our knowledge, except for the increased dosing effect of *RUNX1* in ML-DS, no increased prevalence and no specific mechanism of interaction between altered *RUNX1* and *GATA1* has been described [31, 43–45].

Regarding cytogenetics, we could not affirm an inverse correlation of *RUNX1* mutation and the recurrent CBF-defining abnormalities t(8;21) and inv(16)/t(16;16). Consistent with reports in adult AML, *RUNX1* mutation weakly associated with the unbalanced abnormality −7/7q [13, 16], which has also been described as myelodysplasia related aberration. However, none of the analysed paediatric AML patients had a known history of MDS and further cytogenetic alterations related to myelodysplasia, including −5/5q, were almost absent in the evaluated cohort. In contrast to previous reports, *RUNX1* mutation did not correlate with trisomy +8 [16, 39, 46], trisomy +13 was even absent in the analysed *RUNX1*-mutated paediatric cohort [13, 15, 47]. Distribution of normal and complex karyotype was largely balanced, which in part, agrees with results of a small Japanese paediatric AML cohort, where 6 out of 11 *RUNX1*-mutated patients had normal karyotype, whilst +8 and complex KT were present in one patient [33]. Ultimately, *RUNX1*-mutated paediatric AML did not show a distinct cytogenetic pattern, particularly with respect to the overlap with prognostically favourable subgroup CBF-AML, arguing against *RUNX1* mutation as a distinct AML type in paediatrics.

*RUNX1*-mutated AML completely lacks *KMT2A*r, indicating that *RUNX1* mutation most probably do not contribute to leukaemogenesis in *KMT2A*r AML in paediatrics. In fact, in AML mouse models harbouring *KMT2A*r, *RUNX1* activity mediated leukaemic cell growth and survival [48, 49]. Similarly, *RUNX1* was determined as transcriptional target in *KMT2A::AFF1*, previously known as *MLL::AF4*, AML by Wilkinson et al. [49], suggesting that *RUNX1* activity cooperates with *KMT2A*r to promote AML development. Considering the determined exclusivity of *RUNX1* mutation and *KMT2A*r, this could also be true for paediatric AML.

Given that *RUNX1* mutations alone are highly unlikely to be sufficient to cause complete leukaemic transformation [50], investigation of cooperating mutations is required to further elucidate leukaemic mechanisms in *RUNX1*-mutated AML. Increased number of co-occurring mutations indicates a more complex genetic evolution of *RUNX1*-mutated AML, requiring a broad range of additional mutations.

*FLT3*-ITD was the most common co-occurring mutation and significantly associated with *RUNX1* mutation in paediatric AML, which may suggest cooperativity between these two mutations. An early report indicated a link between *RUNX1* mutation and *FLT3*-ITD contributing to leukaemogenesis in AML M0, in which *RUNX1* mutations predominated [51]. In comparison, *FLT3*-ITD frequently occurred in *RUNX1*-mutated adult AML, albeit evenly distributed and without correlation with *RUNX1* mutation [15, 39], supporting the notion of a cooperation between *RUNX1* mutation and *FLT3*-ITD, which may be specific to paediatric AML [52]. *RUNX1* mutations were almost exclusive of alterations involved in other activated-signalling pathways, namely *NRAS, KRAS* and *KIT*, which in contrast, were relatively common in the wt-cohort. These results may point at a reassortment in the functional group of activated-signalling, shifted towards *FLT3*-ITD, supporting the suggestion, that *RUNX1* mutation preferably contributes to leukaemogenesis by cooperating with the activating *FLT3*-ITD, instead of other activating pathways in paediatric AML. Despite the fact that *FLT3*-ITD has long been known to associate with poor prognosis [53, 54], in the present study, co-occurrence of *FLT3*-ITD with *RUNX1* mutation did not affect outcomes in paediatric AML, which is consistent with results in adult AML [15].

Despite the small number of patients, which makes the accurate assessment of additional genetic associations more difficult, concurrent genetic aberrations occurred more frequently in epigenetic modifiers, with a trend towards *BCORL1*, *EZH2*, and *KDM6A*. A propensity for association was noted for spliceosome *U2AF1*, whereas other spliceosome-related genetic aberrations were absent in *RUNX1*-mutated paediatric AML. In contrast, *RUNX1*-mutated adult AML has been associated with mutations in the spliceosome and cohesion complex, in addition to epigenetic modifiers [13], consistent with those reported in high-risk MDS [55, 56]. This further underlines the heterogeneity in biological properties between adults and paediatric AML.

Consistent with previous studies [12–15, 57], mutations of the transcription factor *NPM1* were completely absent in the *RUNX1*-mutated paediatric cohort. *NPM1* mutation is considered as founding genetic event in AML with certain biological and clinical features, warranting its own entity [52, 58]. The exclusivity of *NPM1* and *RUNX1* mutation in AML might suggest that *RUNX1* mutation shares a similar role with *NPM1* mutations in leukaemia development. In two cases we observed that *RUNX1* mutation overlaps with double mutated *CEBPA* in paediatrics, contradicting the notion that *RUNX1*-mutated AML represents a distinct entity. Thus, according to the results of this cohort, previously reported characteristic features, favouring the concept of considering *RUNX1*-mutated AML as a distinct entity [13, 17], cannot be confirmed in paediatric AML since *RUNX1* mutation lacks these mutational features and overlaps with AML defining mutational patterns.

In the recent ELN-recommendation, *RUNX1* mutation was stated as a late event in leukaemia development [21], which contradicts earlier studies, considering *RUNX1* mutations as early events in leukemognenesis [15, 50]. The observed inconsistency of *RUNX1* mutations in six out of eight paired diagnosis-relapse samples in this paediatric cohort, indicates instability, suggesting that *RUNX1* mutation rather is a late genetic event contributing to advanced clonal evolution. Moreover, the detected high number of additional aberrations accompanying *RUNX1* mutation and the observed overlap with the AML-causing events CBF-fusions or *NPM1* mutation in the assessed paediatric patients, further support the concept that RUNX1 mutations are more likely to contribute to the late-stage leukaemia development.

In this study, *RUNX1* mutation did not show a prognostic significance for outcomes and survival. These results contrast with previous data on *RUNX1* mutation in AML, indicating that *RUNX1* is a negative prognosticator [12–14, 33]. Since some reports combined primary and secondary AML, Quesada *et al*. assessed the prognostic impact of *RUNX1*-mutated de novo AML according to the WHO definition and detected no differences in outcome [57], which is concordant with our findings in primary paediatric AML. In addition, Greif *et al*. proved negative prognostic significance in elderly (> 60 years) cytogenetically normal AML, whereas this could not be confirmed for younger patients [14, 16], suggesting age-dependency of the poor prognostic impact of *RUNX1* mutation. This observation could be strengthened by our results, where *RUNX1* mutation showed no predictive relevance in paediatric AML. However, we did not observe a strong difference when separately assessing age-depending prognostic impact of *RUNX1* mutation in paediatric patients. Since the survival was not affected by *RUNX1* mutation in different risk groups, there seems to be no relevance for including *RUNX1* mutations in the risk stratification of paediatric AML. In terms of indication for SCT, survival was comparable in patients with and without SCT in first CR, suggesting that the presence of *RUNX1* mutation does not require modification in eligibility criteria in paediatric AML.

This analysis uncovers the impact of *RUNX1* mutation in a well characterised paediatric AML cohort and provides new insights into its clinicopathological relevance. Although this study evaluates *RUNX1* mutations in the largest paediatric cohort studied to date, analyses are still limited due to small sample sizes for some analysis-subgroups and the retrospective dataset, necessitating further validation to confirm these observations.

In conclusion, *RUNX1* mutations did not impact outcomes in this paediatric AML cohort, suggesting a clinical impact that differs from that reported in adult AML. Importantly, *RUNX1* mutations lacked clinical characteristics and genetic features, which would justify a separate WHO entity. Therefore, the previously stated provisional WHO entity ‘*RUNX1* mutated AML’, is not appropriate in paediatric AML. The results of this study agree with the recently published fifth edition of the WHO classification which removed *RUNX1* mutations as distinct entities in AML [19].

## Supplementary information

Supplementary Material

## Data Availability

The code used for the t-SNE analysis and visualisation can be provided upon request.

## Data availability

A detailed data sharing statement is provided in the Supplementary Materials.
