# Supplementary material for: RUNX1 mutation has no prognostic significance in paediatric AML: a retrospective study of the AML-BFM study group
Source: Leukemia. 2023 May 15;37(7):1435–43. doi: 10.1038/s41375-023-01919-8 (PMC10317839; doi:10.1038/s41375-023-01919-8)

**Supplementary Appendix**

**RUNX1 mutation has no prognostic significance in paediatric AML: A retrospective study of the AML-BFM study group**

Stephanie Sendker^1^, Amani Awada^1^, Sophia Domagalla^1^, Michael Sendker ^2^, Eser Orhan ^3^, Lina Marie Hoffmeister^1^, Evangelia Antoniou^1^, Naghmeh Niktoreh^1^, Dirk Reinhardt^1^, Nils von Neuhoff^1^ and Markus Schneider^1^*

^1^ Department of Pediatric Hematology and Oncology, University Children’s Hospital Essen, University of Duisburg-Essen, 45147 Essen, Germany

^2^ University of Applied Sciences for Economics and Management (FOM), 20357 Hamburg, Germany

^3^ Centre for Research Acceleration in Pediatrics GmbH, 30175 Hannover, Germany

*Corresponding author: Markus Schneider, Markus.Schneider@uk-essen.de

These supplementary data provide details on used statistical methods, the investigated *RUNX1* mutations, further genetic and clinical data as well as subgroup analysis of outcome and survival and results of the cox regression analysis.

**Table of contents**

[Data Sharing Statements of the AML-BFM Study Group 3](#_Toc129344635)

[Supplementary Table S1a 4](#_Toc129344636)

[Supplementary Table S1b 4](#_Toc129344637)

[Supplementary Table S2a. 6](#_Toc129344638)

[Supplementary Table S2b 7](#_Toc129344639)

[Supplementary Table S2c 9](#_Toc129344640)

[Supplementary Table S3 10](#_Toc129344641)

[Supplementary Table S4a 11](#_Toc129344642)

[Supplementary Table S4b 12](#_Toc129344643)

[Supplementary Table S5a 13](#_Toc129344644)

[Supplementary Table S5b 14](#_Toc129344645)

[Supplementary Table S6 15](#_Toc129344646)

[Supplementary Figure S1 18](#_Toc129344647)

Data Sharing Statements of the AML-BFM Study Group

The AML-BFM Study Group Data Sharing policy describes the release and use of AML-BFM individual subject data for use in research projects in accordance with EU-Directive of Good Clinical Practice, the guidelines of the German Research Foundation (DFG) and the German Society of Pediatric Oncology and Hematology (GPOH). Only data expressly released from the oversight of the relevant AML-BFM Data and Safety Monitoring Committee (DSMC) are available to be shared. Data sharing will ordinarily be considered only after the primary study manuscript is accepted for publication. For phase III trials, individual-level de-identified datasets that would be sufficient to reproduce results provided in a publication containing the primary study analysis can be requested from the AML-BFM data management. Data are available to researchers who wish to analyze the data in secondary studies to enhance the public health benefit of the original work and agree to the terms and conditions of use. For non-phase III trials, data are available following the primary publication. An individual-level de-identified dataset containing the variables analyzed in the primary results paper can be expected to be available upon request. Requests for access to AML-BFM protocol research data should be sent to the AML-BFM Study Group offices. Data are available to researchers whose proposed analysis is found by the AML-BFM research board to be feasible and of scientific merit and who agree to the terms and conditions of use. For all requests, no other study documents, including the protocol, will be made available and no end date exists for requests. In addition to above, release of data collected in a clinical trial conducted under a binding collaborative agreement between AML-BFM Study Group and a pharmaceutical/biotechnology company must comply with the data sharing terms of the binding collaborative/contractual agreement and must receive the proper approvals.

Supplementary Table S1a

Detailed information on the analyzed *RUNX1*-mutated patient samples.

| **Pat No.** | **FAB** | **Status of disease** | **Sex** | **Age** | **Diagnosis (year)** | **AML register** |
| --- | --- | --- | --- | --- | --- | --- |
| 1 | M1 | Initial | M | 8.9 | 2016 | AMLR12 |
| 2 | M2 | relapse | F | 12.1 | 2019 | AMLR17 |
| 3 | M1 | Initial | F | 14.2 | 2018 | AMLS12 |
| 4 | M7 | Initial | M | 2.4 | 2017 | AMLR12 |
| 5 | M1 | Initial | M | 15.6 | 2016 | AMLS12 |
| 6 | M4eo | Initial | M | 14.5 | 2017 | AMLS12 |
| 7 | M2 | Initial | M | 8.5 | 2017 | AMLS12 |
| 8 | M2 | Initial | F | 17.0 | 2021 | AMLR17 |
| 9 | M4 | Initial | F | 14.3 | 2019 | AMLR17 |
| 10 | M1 | Initial | M | 12.8 | 2016 | AMLS12 |
| 11 | M4 | Initial | M | 15.7 | 2021 | AMLR17 |
| 12 | M2 | relapse | M | 12.2 | 2019 | AMLR17 |
| 13 | M4 | Initial | M | 5.1 | 2016 | AMLR12 |
| 14 | M4 | relapse | M | 5.5 | 2016 | AMLR17 |
| 15 | M5 | Initial | F | 14.0 | 2017 | AMLS12 |
| 16 | M2 | relapse | F | 7.5 | 2018 | AMLR17 |
| 17 | M0 | Initial | M | 14.0 | 2016 | AMLS12 |
| 18 | M2 | relapse | M | 16.3 | 2018 | AMLR17 |
| 19 | M2 | Initial | F | 11.7 | 2018 | AMLR17 |
| 20 | M4 | Initial | M | 5.2 | 2015 | AMLR12 |
| 21 | M4 | Initial | M | 16.0 | 2019 | AMLR17 |
| 22 | M5 | Initial | M | 15.4 | 2017 | AMLS12 |
| 23 | M4 | Initial | F | 5.2 | 2019 | AMLR17 |

Abbreviations: FAB, French American British; AMLR12. AML Register 2012; AMLR17, AML Register 2017; AMLS12. AML study 2012; AMLS17, AML study 2017.

Supplementary Table S1b

Detailed information on *RUNX1* mutation types. Locations of mutations are defined according to the *RUNX1* transcript variant 2 (NM_001001890, reference sequence/ Gene bank ID NG_011402. NCBI; [www.ncbi.org](http://www.ncbi.org/)).

| **Pat No.** | **Nucelotide change** | **Amino acid change** | **VAF** | **Reads** | **Exon** | **Domain** | **Type of mutation** |
| --- | --- | --- | --- | --- | --- | --- | --- |
| 1 | *c.623_626delCCATinsACAG* | *p.A208_M209delinsDR* | 9.12 | 581 | 5.1 | n.a. | delins |
| 2 | *c.623_626delinsACAG* | *p.A208_M209delinsDR* | 8.61 | 1092 | 5.1 | n.a. | delins |
|  | c.616C>T | p.R206C | 9.32 | 1095 | 5.1 | n.a. | missense |
| 3 | c.1193dupC | p.P399Afs* | 12.81 | 687 | 6 | RD | frameshift |
| 4 | c.238C>T | p.R80C | 5.39 | 3154 | 2 | RHD | missense |
| 5 | c.669_670ins25 | p.A224Rfs* | 5.27 | 1063 | 5.1 | n.a. | frameshift |
| 6 | c.709dupC | p.Q237Pfs* | 41.23 | 1460 | 5.1 | n.a. | frameshift |
| 7 | c.616C>T | p.R206C | 8.61 | 697 | 5.1 | n.a. | missense |
|  | *c.623_626delCCATinsACAG* | *p.A208_M209delinsDR* | 8.33 | 696 | 5.1 | n.a. | delins |
| 8 | *c.1225_1228delinsCT* | *p.S409Lfs** | 20.56 | 958 | 6.0 | RD | frameshift |
|  | c.293del | p.P98Qfs* | 19.8 | 6145 | 3.0 | RHD | frameshift |
| 9 | c.1126dupT | p.Y376Lfs* | 51.84 | 544 | 6.0 | RD | frameshift |
| 10 | c.389G>T | p.R130I | 39.48 | 7751 | 3.0 | RHD | missense |
| 11 | c.1175_1181dup | p.E395Gfs* | 44.88 | 800 | 6.0 | RD | frameshift |
| 12 | c.265delinsCC | p.F89Pfs* | 22.91 | 1528 | 2.0 | RHD | frameshift |
| 13 | c.623_626delCCATinsACAG | p.A208_M209delinsDR | 6.69 | 1046 | 5.1 | n.a. | delins |
|  | c.616C>T | p.R206C | 6.86 | 1049 | 5.1 | n.a. | missense |
| 14 | c.1273dupG | p.V425Gfs* | 9.66 | 4637 | 6.0 | n.a. | frameshift |
| 15 | c.330_346delTGAAAACTACTCGGCTGinsAAGGATGT | p.D110_E116delins4 | 27.8 | 4605 | 3 | RHD | delins |
| 16 | c.354_364dup | p.A122Efs* | 17.15 | 6158 | 3 | RHD | frameshift |
| 17 | c.538C>T | p.R180W | 11.24 | 5586 | 5.1 | n.a. | missense |
| 18 | c.331G>A | p.E111K | 5.01 | 6444 | 3 | RHD | missense |
| 19 | c.359C>A | p.A120D | 31.38 | 6816 | 3.0 | RHD | missense |
| 20 | c.912_924delCGACCCGCGCCAG | p.D305Sfs* | 15.52 | 2315 | 6.0 | TAD | frameshift |
| 21 | c.521G>A | p.R174Q | 44.53 | 2214 | 4.0 | RHD | missense |
| 22 | c.871_872delTC | p.S291Qfs* | 38.98 | 4418 | 5.2 | TAD | frameshift |
| 23 | c.413_416delGTCG | p.G138Qfs* | 35.65 | 7044 | 3.0 | RHD | frameshift |

Abbreviations: VAF, Variant allele frequency; RHD, Runt homology domain; TAD, transactivation domain (TAD), RD, repression domain.

Supplementary Table S2a.

| **number of additional mutations** | ***RUNX1*wt** | ***RUNX1*mut** | ***p*-value** |
| --- | --- | --- | --- |
|  | **no. (%)** | **no. (%)** |  |
| 0 | 93 (19.8) | 1 (5.6) | 0.22 |
| 1 | 151 (32.3) | 2 (11.1) | 0.05 |
| 2 | 101 (21.6) | 9 (50) | 0.01 |
| 3 | 72 (15.3) | 3 (16.7) | 0.75 |
| 4 | 32 (6.8) | 2 (11.1) | 0.36 |
| > 4 | 22 (4.7) | 1 (5.6) | 0.59 |
| Median | 1.7 | 2.4 | 0.002 |
| Range | 0 - 6 | 0 - 5 |  |

Number of molecular genetic mutations detected by NGS in *RUNX1*-mutated (mut) and wildtype (wt) cohort.

Supplementary Table S2b

Molecular genetic data. Mutational status of 54 leukemia associated genes analyzed by NGS in *RUNX1*-mutated (mut) and *RUNX1* wildtype (wt) cohort.

| **NGS data** | ***RUNX1*wt (n=470)**  **No (%)** | ***RUNX1*mut (n=18)**  **No (%)** | ***p*-value** |
| --- | --- | --- | --- |
| *FLT3*-ITD | 102 (21.7) | 10 (55.6) | 0.002 |
| *NRAS* | 99 (21.1) | 1 (5.6) | 0.14 |
| *WT1* | 55 (11.7) | 4 (22.2) | 0.26 |
| *KIT* | 47 (10.0) | 0 (0) | 0.24 |
| *KRAS* | 46 (9.8) | 0 (0) | 0.4 |
| *NPM1* | 44 (9.4) | 0 (0) | 0.39 |
| *PTPN11* | 34 (7.2) | 1 (5.6) | 1.0 |
| *ASXL1* | 27 (5.7) | 1 (5.6) | 1.0 |
| *CEBPA* | 26 (5.5) | 2 (11.1) | 0.28 |
| monoallelic | 12 (2.6) | 0 (0) |  |
| bimut. | 14 (3.0) | 2 (11.1) |  |
| *TET2* | 19 (4.0) | 1 (5.6) | 0.54 |
| *BCORL1* | 17 (3.6) | 2 (11.1) | 0.17 |
| *CSF3R* | 16 (3.4) | 1 (5.6) | 0.48 |
| *GATA2* | 15 (3.2) | 2 (11.1) | 0.13 |
| *TP53* | 14 (3.0) | 0 (0) | 1.0 |
| *RAD21* | 12 (2.6) | 1 (5.6) | 0.39 |
| *FLT3-TKD* | 12 (2.6) | 0 (0) | 1.0 |
| *IDH2* | 11 (2.3) | 1 (5.6) | 0.4 |
| *EZH2* | 10 (2.1) | 1 (5.6) | 0.37 |
| *CDKN2A* | 10 (2.1) | 0 (0) | 1.0 |
| *IKZF1* | 10 (2.1) | 0 (0) | 1.0 |
| *PHF6* | 9 (1.9) | 1 (5.6) | 0.35 |
| *CUX1* | 9 (1.9) | 0 (0) | 1.0 |
| *IDH*1 | 9 (1.9) | 0 (0) | 1.0 |
| *DNMT3A* | 8 (1.7) | 0 (0) | 1.0 |
| *NOTCH1* | 8 (1.7) | 0 (0) | 1.0 |
| *BCOR* | 7 (1.5) | 0 (0) | 1.0 |
| *ETV6* | 7 (1.5) | 0 (0) | 1.0 |
| *ZRSR2* | 6 (1.3) | 0 (0) | 1.0 |
| *CBL* | 6 (1.3) | 0 (0) | 1.0 |
| *JAK2* | 5 (1.1) | 0 (0) | 1.0 |
| *KDM6A* | 4 (0.9) | 1 (5.6) | 0.17 |
| *STAG2* | 4 (0.9) | 0 (0) | 1.0 |
| *SMC1A* | 4 (0.9) | 0 (0) | 1.0 |
| *U2AF1* | 3 (0.6) | 1 (5.6) | 0.15 |
| *SRSF2* | 2 (0.4) | 0 (0) | 1.0 |
| *SMC3* | 2 (0.4) | 0 (0) | 1.0 |
| *BRAF* | 2 (0.4) | 0 (0) | 1.0 |
| *MPL* | 2 (0.4) | 0 (0) | 1.0 |
| *PTEN* | 2 (0.4) | 0 (0) | 1.0 |
| *ATRX* | 2 (0.4) | 0 (0) | 1.0 |
| *PDGFRA* | 1 (0.2) | 0 (0) | 1.0 |
| *ABL1* | 1 (0.2) | 0 (0) | 1.0 |
| *MYD88* | 1 (0.2) | 0 (0) | 1.0 |
| *SETBP1* | 1 (0.2) | 0 (0) | 1.0 |
| *CALR* | 1 (0.2) | 0 (0) | 1.0 |
| *FBXW*7 | 1 (0.2) | 0 (0) | 1.0 |
| *SF3B1* | 0 (0) | 0 (0) |  |
| *GATA1* | 0 (0) | 0 (0) |  |
| *GNAS* | 0 (0) | 0 (0) |  |
| *JAK3* | 0 (0) | 0 (0) |  |
| *NOTCH1* | 0 (0) | 0 (0) |  |
| *CBLB* | 0 (0) | 0 (0) |  |
| *CBLC* | 0 (0) | 0 (0) |  |
| *RUNX1* | 0 (0) | 18 (100) |  |
| no mutation | 89 (18.9) | 0 (0) |  |

Supplementary Table S2c

Functional group of genetic mutation. Distribution of mutated genetic groups in *RUNX1*-mutated (mut) and wildtype (wt) cohort.

| **functional group of additional mutation** | ***RUNX1*wt** | ***RUNX1*mut** | ***p*-value** |
| --- | --- | --- | --- |
|  | **no. (%)** | **no. (%)** |  |
| Spliceosome | 11 (2) | 1 (3) | 0.38 |
| Epigenetic modifier | 76 (10) | 7 (22) | 0.13 |
| Tumor suppressor | 88 (12) | 5 (16) | 0.38 |
| Transcription factors | 111 (15) | 4 (13) | 0.91 |
| Cohesin | 22 (3) | 1 (3) | 0.89 |
| DNA methylation | 47 (6) | 1 (3) | 0.88 |
| Activated signaling | 371 (50) | 13 (41) | 0.71 |
| Others | 18 (2) | 0 (0) | 0.4 |

Supplementary Table S3

Response, outcome and SCT. Distribution of response rates, prognostic endpoints and stem cell transplantation (SCT) at different timepoints in RUNX1 mutated (mut) and wildtype (wt) cohort.

| **Characteristics** | | ***RUNX1*wt (n=470)** | ***RUNX1*mut (n=18)** | ***p*-value** |
| --- | --- | --- | --- | --- |
| Number | | 470 (96 %) | 18 (4 %) |  |
| SCT | no SCT | 288 (61 %) | 11 (61 %) | 0.97 |
|  | SCT | 182 (39 %) | 7 (39 %) |  |
|  | SCT CR1 | 108 (23 %) | 4 (22 %) | 1.0 |
|  | SCT CR2 | 57 (12 %) | 2 (11 %) | 1.0 |
|  | SCT NR | 17 (4 %) | 1 (6 %) | .5 |
| Outcome | Death | 94 (20 %) | 1 (6 %) | 0.22 |
|  | Events | 174 (37 %) | 6 (33 %) | 0.75 |
|  | Relapse | 108 (23 %) | 3 (17 %) | 0.78 |
| Response | CR (rate %) | 393 (84 %) | 17 (94 %) | 0.33 |
|  | NR (rate %) | 57 (12 %) | 1 (6 %) | 0.71 |
|  | ED | 20 (4 %) | 0 (0 %) | 1.0 |

Supplementary Table S4a

Univariable Cox regression analysis according to status of *RUNX1* mutation in pediatric AML.

| **variable** | **OS** | | | | **EFS** | | | |
| --- | --- | --- | --- | --- | --- | --- | --- | --- |
|  | ***p*-value** | **HR** | **95 % CI** | | ***p*-value** | **HR** | **95 % CI** | |
| ***RUNX1*** | 0.17 | 4,32 | 0.60 | 30.97 | 0.55 | 0.78 | 0.34 | 1.76 |
| **gender** | 0.11 | 0.72 | 0.48 | 1.08 | 0.13 | 0.80 | 0.59 | 1.07 |
| **age** | 0.43 | 0.99 | 0.95 | 1.02 | 0.66 | 1.01 | 0.98 | 1.03 |
| **risk*** | 0.00 | 0.01 | 0.00 | 0.06 | 0.00 | 0.10 | 0.04 | 0.29 |
| ***FLT3*-ITD** | 0.82 | 0.94 | 0.58 | 1.53 | 0.44 | 1.14 | 0.82 | 1.60 |
| ***WT1*** | 0.73 | 1.11 | 0.62 | 1.99 | 0.13 | 1.37 | 0.92 | 2.05 |
| ***NRAS*** | 0.02 | 0.47 | 0.25 | 0.89 | 0.17 | 0.76 | 0.52 | 1.12 |
| ***KMT2A*r** | 0.01 | 1.81 | 1.15 | 2.85 | 0.21 | 1.26 | 0.88 | 1.79 |
| **Complex KT** | 0.03 | 1.69 | 1.06 | 2.69 | 0.22 | 1.26 | 0.87 | 1.80 |
| **Normal KT** | 0.70 | 0.91 | 0.58 | 1.44 | 0.45 | 1.13 | 0.82 | 1.56 |
| **FAB M4** | 0.07 | 1.54 | 0.97 | 2.47 | 0.04 | 1.45 | 1.02 | 2.06 |
| **SCT** | 0.06 | 1.47 | 0.98 | 2.20 | < 0.001 | 3,47 | 2.55 | 4,72 |
| **blasts BM** | 0.10 | 1.01 | 1.00 | 1.02 | 0.64 | 1.00 | 1.00 | 1.01 |
| **Add. mut.** | 0.39 | 0.94 | 0.80 | 1.09 | 0.68 | 1.02 | 0.92 | 1.14 |

Abbreviation: OS, Overall survival; EFS, Event-free survival; HR, hazard ratio. CI, confidence interval. Mut, mutation. Wt, wildtype. KT, karyotype, SCT, Stem cell transplantation; BM, bone marrow; Add. mut., number of co-occurring mutations.

Supplementary Table S4b

Multivariable Cox regression analysis according to status of *RUNX1* mutation in pediatric AML. Age at diagnosis, risk group adapted to survival in the investigated cohort (risk), presence of *FLT3*-ITD, Stem cell transplantation (SCT) and number of co-occurring mutations (add. mut.) were considered as covariates.

Abbreviation: OS, Overall survival; EFS, Event-free survival; HR, hazard ratio. CI, confidence interval. Mut, mutation. Wt, wildtype.

| **variable** | **OS** | | | | **EFS** | | | |
| --- | --- | --- | --- | --- | --- | --- | --- | --- |
|  | ***p*-value** | **HR** | **95 % CI** | | ***p*-value** | **HR** | **95 % CI** | |
| ***RUNX1*** | 0.16 | 0.23 | 0.03 | 1.70 | 0.47 | 0.73 | 0.31 | 1.71 |
| **age** | 0.63 | 0.99 | 0.96 | 1.03 | 0.97 | 1.00 | 0.97 | 1.03 |
| **risk** | <0.001 | 0.01 | 0.00 | 0.06 | <0.001 | 0.10 | 0.03 | 0.29 |
| ***FLT3*-ITD** | 0.86 | 0.95 | 0.56 | 1.61 | 0.87 | 1.03 | 0.72 | 1.48 |
| **SCT** | 0.42 | 0.82 | 0.50 | 1.33 | 0.00 | 3.37 | 2.36 | 4.83 |
| **Add. mut.** | 0.82 | 1.02 | 0.87 | 1.20 | 0.34 | 1.06 | 0.95 | 1.18 |

Supplementary Table S5a

Subgroup analysis of overall survival (OS) and event-free survival (EFS) in different patient groups, selected for accompanying mutations (less or equal to 2 vs. more than 2 mutations), age (younger or equal to 12 or older than 12 years), stem cell transplantation (SCT) in first complete remission (CR1) and presence of *FLT3*-ITD.

| **Variable** | **RUNX1 status** | **OS** | | **EFS** | |
| --- | --- | --- | --- | --- | --- |
|  |  | ***p*-value** | **5-y OS (%)** | ***p*-value** | **5-y EFS (%)** |
| ≤2 mutations | wt | 0.26 | 77.0±2.5 | 0.46 | 61.4±2.8 |
|  | mut |  | 100.0±0 |  | 80.0±17.9 |
| >2 mutations | wt | 0.24 | 74.9±5.0 | 0.48 | 49.3±6.3 |
|  | mut |  | 90.9±8.7 |  | 54.2±16.2 |
| age < 12 years | wt | 0.58 | 77.7±2.8 | 0.95 | 62.3±3.2 |
|  | mut |  | 63.5±16.9 |  | 53.3±17.3 |
| age ≥ 12 years | wt | 0.11 | 76.9±3.5 | 0.36 | 54.5±4.3 |
|  | mut |  | 100±0 |  | 71.6±14 |
| no SCT CR1 | wt | 0.17 | 76.5±2.6 | 0.46 | 58.8±3.1 |
|  | mut |  | 100±0.0 |  | 70.7±12.4 |
| SCT CR1 | wt | 0.91 | 77.0±4.4 | 0.98 | 57.1±5.2 |
|  | mut |  | 75.0±21.7 |  | 60±21.9 |
| No *FLT3*-ITD | wt | 0.75 | 76.9±2.5 | 0.41 | 59.9±3.0 |
|  | mut |  | 80.0±12.6 |  | 70.0±14.5 |
| *FLT3*-ITD mut | wt | 0.42 | 78.8±4.3 | 0.68 | 56.6±5.5 |
|  | mut |  | 80.0±17.9 |  | 57.1±16.4 |

Supplementary Table S5b

Subgroup analysis of overall survival (OS) and event-free survival (EFS) according to risk group allocation into standard-risk (SR), intermediate-risk (IR) and high-risk (HR).

| **Variable** | ***RUNX1*** | **OS** | | **EFS** | |
| --- | --- | --- | --- | --- | --- |
|  |  | ***p*-value** | **5-y OS (%)** | ***p*-value** | **5-y EFS (%)** |
| **SR (n=123)** | **wt** | 0.58 | 92.7±2.5 | 0.81 | 73.2±4.4 |
|  | **mut** |  | 100.0±0 |  | 75.0±21.7 |
| **IR (n=207)** | **wt** | 0.14 | 78.2±3.2 | 0.72 | 57.1±4.3 |
|  | **mut** |  | 100.0±0 |  | 70.0±14.5 |
| **HR (n=123)** | **wt** | 0.94 | 59.8±5 | 0.57 | 44.4±4.9 |
|  | **Mut** |  | 50.0±20.4 |  | 37.5±28.6 |

Supplementary Table S6

Tabular overview of clinical, morphological, immunophenotypic and genetic characteristics in pediatric AML patients with relapsed disease during leukemic evolution from initial diagnosis to relapse. RUNX1 mutational status was inconsistent in six patient samples. Five patients had RUNX1 mutation at relapse only, in one patient, RUNX1 mutation was detectable at initial diagnosis but got lost during leukemic development into an early relapse, whilst in two patients, RUNX1 mutation was present at initial diagnosis and relapse.

| **Patient group** | **Pat No.** | **Timepoint of RUNX1 mutation** | **Status of disease** | **Age** | **Study** | **Risk^*^** | **Morphology** | | | | | | | **Immunophenotype changes** | **Cytogenetic changes** | **Mutation changes** | **Response** | | |
| --- | --- | --- | --- | --- | --- | --- | --- | --- | --- | --- | --- | --- | --- | --- | --- | --- | --- | --- | --- |
|  |  |  |  |  |  |  | **FAB** | **CNS** | **Auer roods** | **POX [%]** | **EST [%]** | **Blasts Bm [%]** | **Blasts Pb [%]** |  |  |  | **Response** | **SCT** | **Death** |
| **RUNX1mut at relapse (n=5)** | 2 | relapse | initial | 12.1 | AMLR17 | IR | M2 | No | Yes | 80 | neg | 56 | 68 | CD19 p+, TdT w+ | l.o. del(16)(q21q23)[5]/46,XX,del(2)(p21),add(17)(p12)[3]/46,XX[8], g.o. +21 | g.o. RUNX1, l.o. NRAS | CR | 0 | 0 |
|  |  |  | relapse | 13.3 |  |  | M2 | No | Yes | 91 | neg | 74 | 1 |  |  |  | CR | SCT CR |  |
|  | 12 | relapse | initial | 12.2 | AMLR17 | IR | M2 | No | yes | 50 | neg | 80 | 21 | CD15 -, CD11b -, CD64 - | g.o. 48,idem,+4 [4] | g.o. RUNX1, WT1, l.o.NRAS | CRp | 0 | 0 |
|  |  |  | 1st relapse | 14.1 |  |  | M2 | No | No | 5 | neg | 45 | 13 |  |  |  | CRp | SCT CR |  |
|  | 14 | relapse | initial | 5.5 | AMLR17 | HR | M4 | Yes | Yes | 20 | 40 | 74 | 46 | CD19 p+ | l.o. add(4)(p16), t(13;17)(p11Mp11),-15,+mar[6]/46,XY[9], g.o. t(2;12)(p12;p12)[15] | g.o. TET2, RUNX1 | NR | SCT CR | 1 |
|  |  |  | 1st relapse | 6.8 |  |  | M4 | No | No | n.a.^1^ | n.a.^1^ | 20 | 3 |  |  |  | CR | SCT boost |  |
|  |  |  | 2nd relapse | 8.3 |  |  | M4 | Yes | No | neg | 60 | 68 | 74 | CD13 +, CD34 - | g.o t(2;12)(p12;p12) | g.o. FLT3-ITD, l.o. TET2 | NR | - |  |
|  | 16 | relapse | initial | 7.5 | AMLR17 | HR | M1 | No | No | neg | neg | 54 | 36 | none | g.o. t(3;12)(q13;q23)[6]/45,idem,dic(11;?)(p11;?)[8]/46,idem,der(11)add(11)(p14)add(11)(q14),+mar[3]//46,XX[2], l.o. t(9;22)(q34q11)[9] | g.o. PTEN, RUNX1 | CR | SCT CR | 1 |
|  |  |  | 1st relapse | 10.1 |  |  | M1 | No | No | n.a.^1^ | n.a.^1^ | 8 | 1 |  |  |  |  | SCT NR |  |
|  | 18 | relapse | initial | 16.3 | AMLR12 | SR | M2 | No | Yes | 98 | neg | 78 | 70 | none | g.o. inv(3)(p22q13),+8,del(9)(q21q31),+13[3] | g.o. RUNX1, CSF3R | CR | - | 0 |
|  |  |  | relapse | 17.4 | AMLR17 |  | M2 | No | Yes | n.a.^1^ | n.a.^1^ | 13 | 2 |  |  |  | CRp | SCT CR |  |
|  |  |  | isolated CNS relapse | 19.3 |  |  | M2 | Yes | No | n.a.^2^ | n.a.^2^ | 0 | 0 | CD79a p+ | n.a. | n.a. | CR | SCT CR |  |
| **RUNX1mut at initial diagnosis (n=1)** | 13 | initial | initial | 5.1 | AMLR12 | IR | M4 | No | No | 6 | 14 | 92 | 81 | none | none | l.o. FLT3 ITD, RUNX1, TET2 | CR | - | 0 |
|  |  |  | relapse | 5.5 |  |  | M4 | No | No | n.a. | n.a. | 70 | 1 |  |  |  | CRp | SCT CR |  |
| **RUNX1mut at initial diagnosis and relapse (n=2)** | 20 | initial & relapse | initial | 5.2 | AMLR12 | HR | M4 | No | No | 10 | 100 | 73 | 9 | none | none, besides additional unsuspicious, female chromosome set due to HSCT | g.o. CSF3R, l.o. FLT3 TKD | CR | SCT CR | 0 |
|  |  |  | relapse | 10.3 |  |  | M4 | No | No | neg | 40 | 25 | 0 |  |  |  | CRp | SCT CR |  |
|  | 22 | initial & relapse | initial | 15.4 | AMLS12 | IR | M5 | Yes | No | neg | 73 | 97 | 3 | none, aberrant expression of CD19 | none | none | CR | - | 0 |
|  |  |  | relapse | 16.6 |  |  | M5 | No | No | neg | n.a. | 23 | 6 |  |  |  | CRp | SCT CR |  |

Abbreviation: Mut, mutation; SR, standard-risk; IR, intermediate-risk; HR, high-risk; FAB, French-American-British AML classification; CNS, central nervous system involvement; POX, Peroxidase positivity; EST, Esterase positivity; BM, bone marrow; Pb, peripheral blood; CD, cluster of differentiation; +, positive, - negative; p+, partial positive; g.o., gain of; l.o., loss of; CR, complete response; CRp, partial response; NR, no response; SCT, Stem cell transplantation.

*Risk allocation at initial diagnosis according to the AML Register 2012 and 2017. No Re-stratification was conducted. ^1^no cytochemistry available due to low blast count. ^2^No cytochemistry performed due to isolated CNS relapse.

Supplementary Figure S1

t-Distributed Stochastic Neighbor Embedding (tSNE) projection, illustrating and clustering RUNX1-mutated samples (red) and samples of the wildtype (wt) cohort (blue) of the studied pediatric AML cohort. In comparison, core binding factor (CBF) AML (yellow) are labeled separately, to illustrate a well definable cluster. In three cases, *RUNX1* mutation overlap with CBF (dark red). 2D visualization is created with Seaborn, a Python visualization library based on matplotlib (Python 3.9.13, Jupyter Notebook 6.4.12).


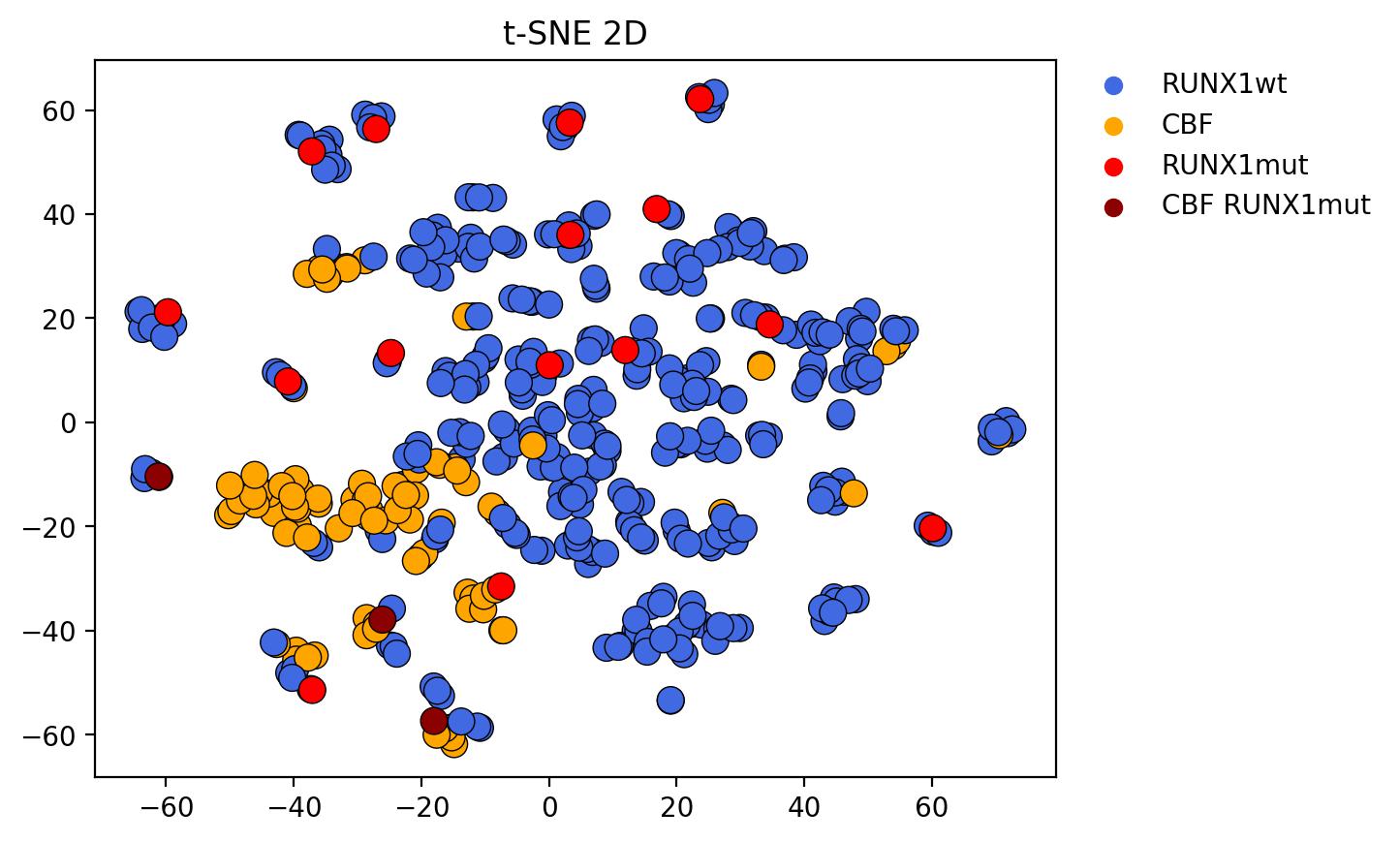

Supplement: Supplementary file 1 — Supplementary Material [file 41375_2023_1919_MOESM1_ESM.docx]
